# Synthesis, Structure, and Characterization of 4,4′-(Anthracene-9,10-diylbis(ethyne-2,1-diyl))bis(1-methyl-1-pyridinium) Bismuth Iodide (C_30_H_22_N_2_)_3_Bi_4_I_18_, an Air, Water, and Thermally Stable 0D Hybrid Perovskite with High Photoluminescence Efficiency

**DOI:** 10.1021/acs.cgd.2c01005

**Published:** 2022-11-22

**Authors:** Lorenza Romagnoli, Andrea D’Annibale, Elena Blundo, Antonio Polimeni, Alberto Cassetta, Giuseppe Chita, Riccardo Panetta, Andrea Ciccioli, Alessandro Latini

**Affiliations:** † Dipartimento di Chimica, Sapienza Università di Roma, Piazzale Aldo Moro 5, 00185Roma, Italy; ‡ Dipartimento di Fisica, Sapienza Università di Roma, Piazzale Aldo Moro 5, 00185Roma, Italy; § Consiglio Nazionale delle Ricerche, Istituto di Cristallografia, Sede Secondaria di Trieste, Area Science Park − Basovizza, Strada Statale 14, km 163.5, 34149Trieste, Italy; ∥ Ispa - Istituto Sperimentale Problematiche Ambientali, Via San Nicandro snc, 03042Atina, FR, Italy

## Abstract

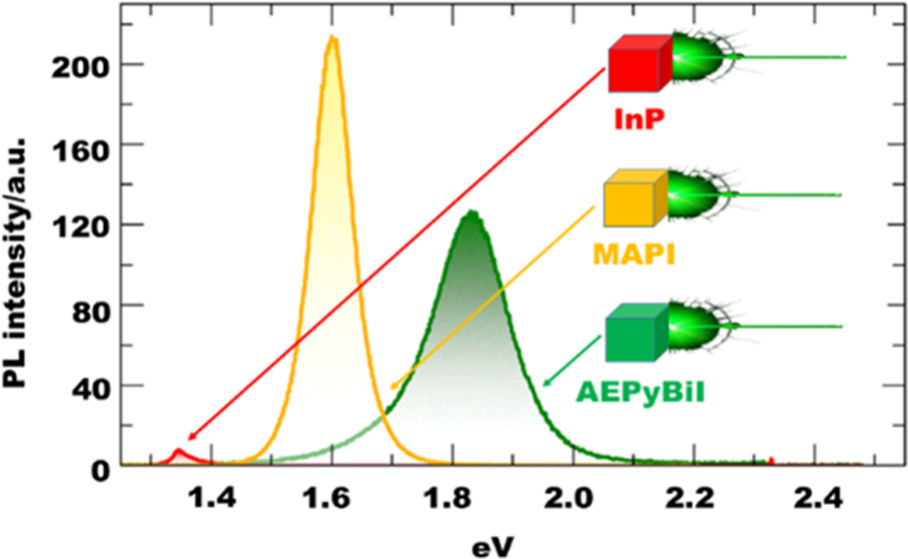

4,4′-(Anthracene-9,10-diylbis(ethyne-2,1-diyl))bis(1-methyl-1-pyridinium) bismuth iodide (C_30_H_22_N_2_)_3_Bi_4_I_18_ (AEPyBiI) was obtained as a black powder by a very simple route by mixing an acetone solution of BiI_3_ and an aqueous solution of C_30_H_22_N_2_I_2_. This novel perovskite is air and water stable and displays a remarkable thermal stability up to nearly 300 °C. The highly conjugated cation C_30_H_22_N_2_^2+^ is hydrolytically stable, being nitrogen atoms quaternarized, and this accounts for the insensitivity of the perovskite toward water and atmospheric oxygen under ambient conditions. The cation in aqueous solution is highly fluorescent under UV irradiation (emitting yellow-orange light). AEPyBiI as well is intensely luminescent, its photoluminescence emission being more than 1 order of magnitude greater than that of high-quality InP epilayers. The crystal structure of AEPyBiI was determined using synchrotron radiation single-crystal X-ray diffraction. AEPyBiI was extensively characterized using a wide range of techniques, such as X-ray powder diffraction, diffuse reflectance UV–vis spectroscopy, Fourier transform infrared (FTIR) and Raman spectroscopies, thermogravimetry-differential thermal analysis (TG-DTA), elemental analysis, electrospray ionization mass spectroscopy (ESI-MS), and photoluminescence spectroscopy. AEPyBiI displays a zero-dimensional (0D) perovskite structure in which the inorganic part is constituted by binuclear units consisting of two face-sharing BiI_6_ octahedra (Bi_2_I_9_^3–^ units). The C_30_H_22_N_2_^2+^ cations are stacked along the *a*-axis direction in a complex motif. Considering its noteworthy light-emitting properties coupled with an easy synthesis and environmental stability, and its composition that does not contain toxic lead or easily oxidable Sn(II), AEPyBiI is a promising candidate for environmentally friendly light-emitting devices.

## Abstract 2

A highly photoluminescent, water-stable bismuth iodide perovskite with a highly conjugated quaternary ammonium cation was synthesized. Its crystal structure was resolved and fully characterized. The perovskite displays a photoluminescence intensity comparable to that of methylammonium lead iodide in the same experimental conditions, without the toxicity problems associated to the use of lead and the water sensitivity problems of the commonly used methylammonium- and formamidinium-based perovskites.

## Introduction

Hybrid metal halide perovskites (HMHPs) are intensely studied materials because of their peculiar photophysical properties and ease of synthesis, which sets them a class apart compared to conventional semiconductors. The largest amount of research is devoted to 3D perovskites, containing small cations, especially methylammonium, formamidinium and cesium, which display remarkable photovoltaic conversion efficiencies in laboratory-scale devices.^1^

Photovoltaics is undoubtedly the technologically and socially most relevant among the research fields involving HMHPs,^2,3^ but there is a growing interest in other applications, which make use of the electronic and photophysical properties of these materials,^4^ especially for light-emitting devices and photodetectors.^5−8^

HMHPs possess a wide range of desirable properties as semiconductors, i.e., easily variable band gap, easy synthesis, solution processability, no need for the same strict purity requirements of conventional semiconductors, no need for high-temperature processes, and no need for highly sophisticated and very expensive fabrication equipments.^9^

In spite of all their useful properties, it is quite surprising that until now no commercial devices of any type that use HMHPs are available. This is mostly due to their lack of stability toward environmental agents (H_2_O and O_2_) and their limited thermal stability.

This lack of chemical and thermal stability is due to the Brønsted acidity of the organoammonium cations typically used.^10−13^ An effective method demonstrated to suppress the sensitivity to water and improve the thermal stability is the use of quaternary ammonium cations,^14^ which lack hydrolysable acidic protons.

In addition to the above problems, there are also specific problems for both Pb and Sn, the most intensely used metals in HMHPs. In the case of lead, its toxicity poses safety and environmental concerns, while in the case of tin, an additional stability problem arises due to the strong tendency of tin(II) to be oxidized to tin(IV) by atmospheric oxygen,^15^ thus complicating device manufacturing because of the need of operating in oxygen-free environments.

To eliminate lead from the composition of HMHPs, in addition to tin, other elements have been investigated, especially Ge^2+^, Sb^3+^, Bi^3+^, and Cu^2+^.^16^ Bi^3+^ is perhaps the most intensely studied for its lack of toxicity,^17^ but the performances of HMHPs containing Bi in photovoltaic devices are quite modest due to the larger band gap of Bi-HMHPs compared to their Pb counterparts.^17^

Although not particularly suitable for photovoltaics, the Bi-HMHPs reported in the literature show interesting photophysical properties, and very efficient light-emitting materials have been successfully prepared and characterized.^18,19^ Although to a lesser degree, Bi-HMHPs suffer from the same instability issues as their Pb counterparts.^20^

**Figure 1:**
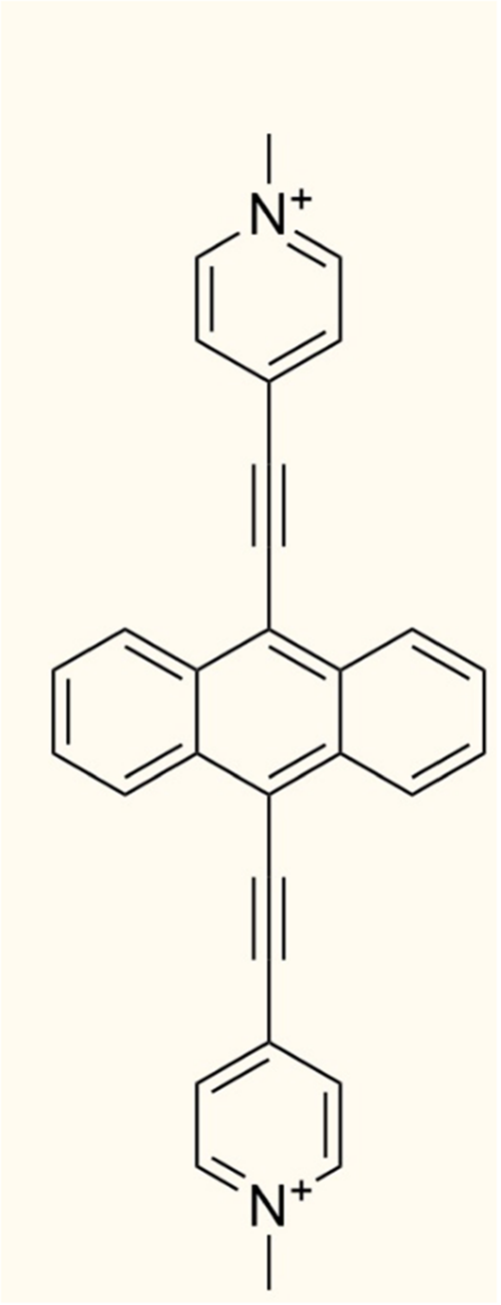
Structure of the AEPy^2+^ cation.

As previously written, quaternary ammonium cations have been demonstrated as a valid strategy to improve the Pb-HMHPs stability, both from thermal and chemical viewpoints. In addition to the stability improvement, quaternary ammonium cations possessing an electronic structure with extended conjugation display novel and potentially useful photophysical properties due to the interaction of the electronic structure of the organic cation with that of the inorganic sublattice.^21^ These interactions, which do not occur in HMHPs with “silent” cations such as methylammonium and formamidinium, open a vast range of possibilities with new, application-tailored properties. In our previous papers,^22,23^ we demonstrated the validity of this approach in the case of phenylviologen lead iodide, whose optoelectronic properties stem from the electronic interaction between the organic cations and the inorganic sublattice. The validity of the approach was demonstrated also by other groups.^24−26^ The material displays excellent thermal and chemical stability as well as intense photoluminescence that may potentially be used for light-emitting devices. These devices, differently from those using conventional semiconductors, may be fabricated using solution-based processes that are considerably less expensive than those used for all-solid-state devices. Considering the promising results obtained in lead-based compounds, we decided to try to develop more environmentally benign alternatives based on nontoxic bismuth, as done previously in the literature for silent cations.^17,20^ In addition to that, we wanted to test cations with a more extended conjugation compared to viologens, but without the need of very complex and expensive synthetic protocols. In this respect, the versatility of 4-bromopyridine in building highly conjugated cations through relatively simple Sonogashira coupling^27^ offers the possibility to obtain a wide variety of organic salts to be reacted with bismuth iodide to obtain the corresponding perovskite. We tried this approach using as a spacer between the *para*-ethynylpyridinium moieties, one of the simplest polycyclic aromatic groups, the 9,10-antharcenyl, and the methyl group to quaternarize the pyridinic nitrogen atoms. We obtained an intensely fluorescent quaternary ammonium cation, namely, 4,4′-(anthracene-9,10-diylbis(ethyne-2,1-diyl))bis(1-methyl-1-pyridinium) (AEPy^2+^, structure in Figure 1 and fluorescence in Figures S1 and S2 of the Supporting Information), which can be easily combined with BiI_3_ to yield a new Bi-HMHP, which is here presented together with its complete structural and physicochemical characterization. The material is air and water insensitive and thermally very stable, as expected for a quaternary ammonium perovskite and shows an intense photoluminescence in the visible range.

## Results and Discussion

### Synthesis

**Figure 2:**
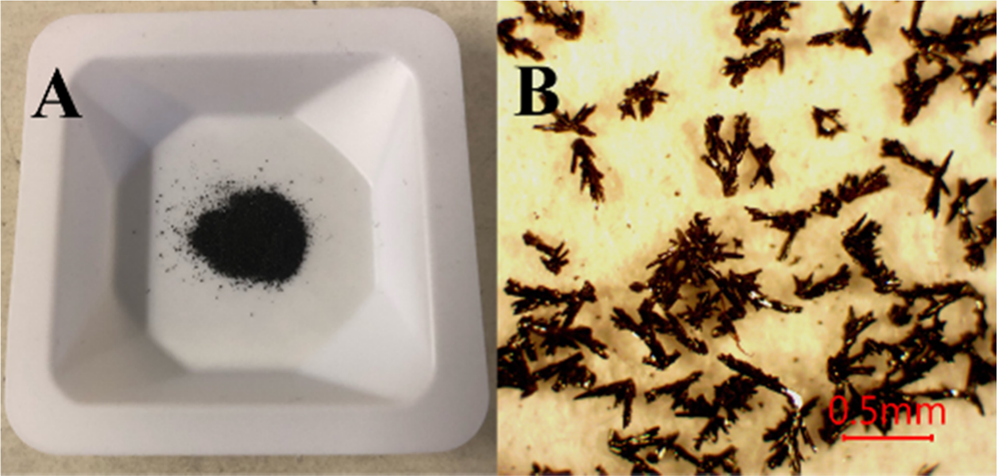
(A) AEPyBiI powder. (B) optical microscopic image of the AEPyBiI powder taken with 4× magnification in reflected light.

**Figure 3:**
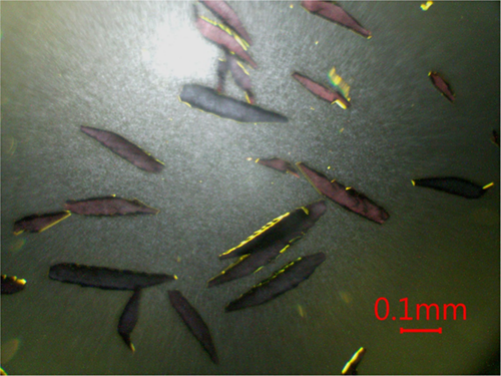
Optical microscopic image of AEPyBiI single crystals taken with 10× magnification with polarized transmitted light and crossed polarizers.

AEPyBiI was prepared by mixing solutions of BiI_3_ and AEPyI_2_
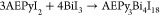
A typical synthesis is described as follows. In a glass vial, 50 mg (0.0753 mmol) of AEPyI_2_ is dissolved in 4 mL of distilled H_2_O; in another vial, 59 mg (0.100 mmol) of BiI_3_ is dissolved in 12 mL of acetone. The first solution, containing the organic salt, is then slowly added, dropwise, to the second; instantaneously, a dark-brown solid starts to precipitate. After the addition is complete, the mixture is vigorously shaken and left undisturbed for 24 h. After that, the dark solid is filtered under suction and washed with several portions of a 3:1 acetone/water mixture. It is then dried for 2 h under suction to yield 89 mg of crude product. The precipitate is subsequently dissolved in 45 mL of *N*,*N*-dimethylformamide (DMF). The dark red solution is filtered over a 0.45 μm PTFE syringe filter and exposed to CH_2_Cl_2_ vapors for 72 h in a closed container at room temperature. After vacuum filtration, the product is washed with multiple portions of CH_2_Cl_2_ and allowed to dry for 1 h under suction to yield 51 mg of pure AEPy_3_Bi_4_I_18_ as black crystals (47%) (Figure 2A,B). Single crystals suitable for structure determination by X-ray diffraction were grown by exposing saturated solutions of AEPy_3_Bi_4_I_18_ in DMF in silanized glass vials to CH_2_Cl_2_ vapors, in a closed vessel, for 72 h at 22 °C (Figure 2). The crystals in Figure 3, observed with polarized light and crossed polarizers, appear of homogeneous color or homogeneously dark as expected for anisotropic (i.e., noncubic) single crystals.

### Structure, Composition, and Stability

AEPyBiI forms dark elongated platelet-shaped crystals with monoclinic structure and the space group **P**21/**n**.

The details of the crystal structure, determined by X-ray diffraction, are summarized in Table 1.

**Table 1:** Crystal Structure Data of AEPyBiI

crystal data	
chemical formula	Bi_2_ I_9_, C_30_ H_21_ N_2_, C_15_ H_11_ N, C_3_ H_7_ N O
*M*_r_	2247.89
crystal system, space group	monoclinic, **P**21/**n**
temperature (K)	298
*a*, *b*, *c* (Å)	10.958 (2), 33.008 (7), 17.070 (3)
α, β, γ (°)	90, 106.42 (3), 90
*V* (Å^3^)	5922 (2)
*Z*	4
radiation type	synchrotron, λ = 0.70000 Å
μ (mm^–1^)	10.26
crystal size (mm)	0.07 × 0.05 × 0.05
data collection	
diffractometer	synchrotron
absorption correction	empirical (using intensity measurements)
SADABS	
no. of measured, independent and observed [*I* > 2.0σ(*I*)] reflections	69652, 10842, 10412
*R*_int_	0.043
θ_min_ = 1.2°	θ_max_ = 24.9°
refinement	
*R*[*F*^2^ > 2σ(*F*^2^)], *w*R**(*F*^2^), *S*	0.042, 0.112, 1.12
no. of reflections	10842
no. of parameters	551
H-atom treatment	hydrogen site location: inferred from neighboring sites
Δρ_max_, Δρ_min_ (e Å^–3^)	1.98, −1.41

**Figure 4:**
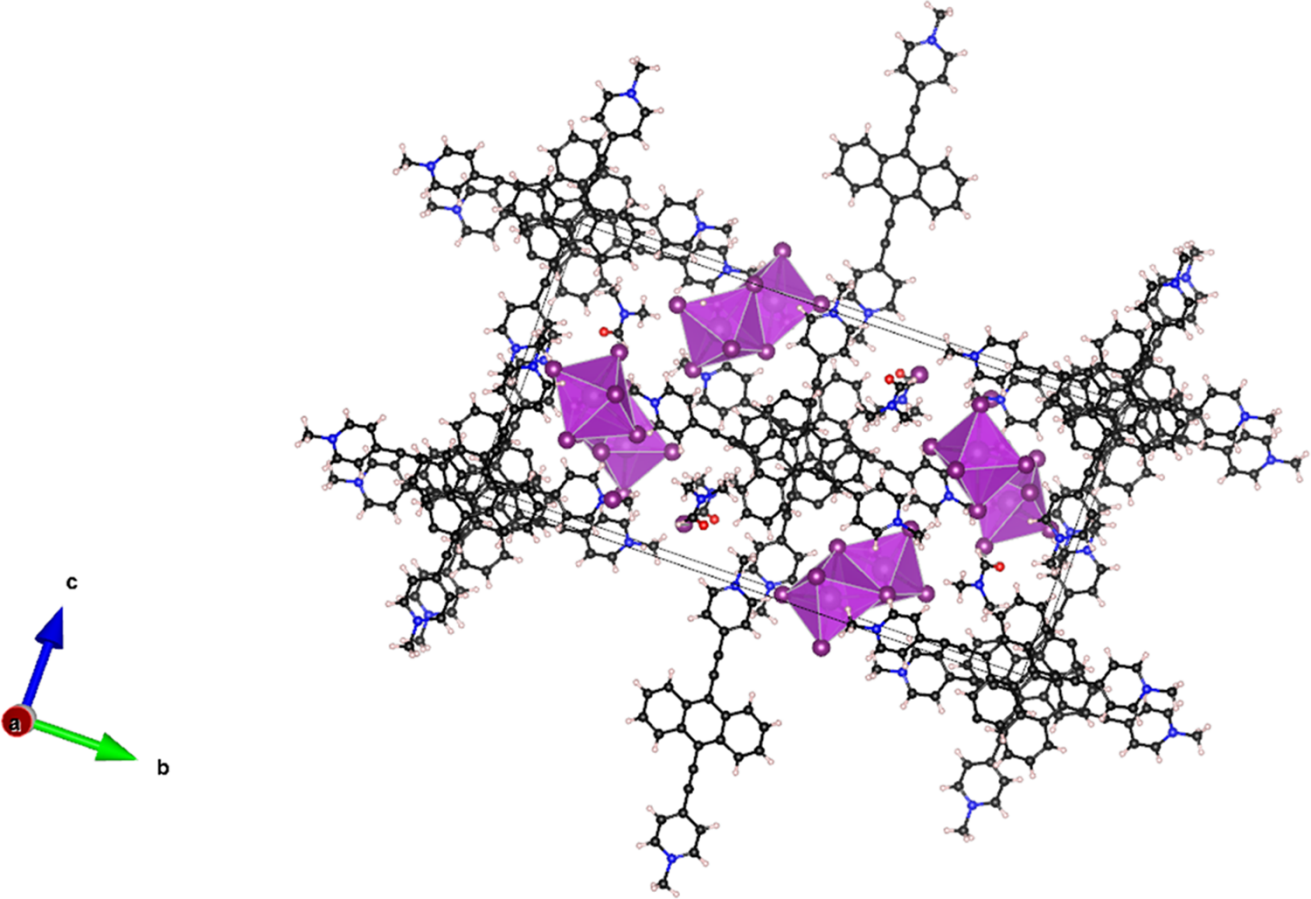
Unit cell of AEPyBiI seen along the *a* crystallographic axis. Black: carbon; blue: nitrogen; white: hydrogen; purple: iodine. Bismuth atoms are the spherical shadows inside the face-sharing octahedra.

A projection of the unit cell is shown in Figure 4.

The structure of AEPyBiI is typical of zero-dimensional (0D) perovskites such as methylammonium bismuth iodide (CH_3_NH_3_)_3_Bi_2_I_9_ and cesium bismuth iodide Cs_3_Bi_2_I_9_ containing isolated Bi_2_I_9_ polyhedra consisting in two BiI_6_^3–^ octahedra sharing one face.^28,29^ The asymmetric unit of the AEPyBiI crystallographic structure includes a Bi_2_I_9_^3–^ anion, a disordered DMF molecule, and one full plus half of AEPy^2+^ cations, with the second half of the incomplete AEPy^2+^ being generated by a center of symmetry lying on the geometric center of the anthracene moiety. A complete representation with the labeling scheme adopted is depicted in Figure S3. The two cations in the asymmetric units are rotated by 90° with respect to each other as represented in Figure S4. AEPy^2+^ cations give rise to a complex interaction network in the crystal. Indeed, AEPy^2+^ cations are stacked together along the *a*-direction according to an ABA repeating motif, where A indicates the AEPy^2+^ cation fully contained in the asymmetric unit and B indicates the AEPy^2+^ half cation observed in the asymmetric unit (see Figure S4). The ABA units extend along the *a*-direction according to the (ABA)(ABA)··· repeating scheme (Figure S5). A and B molecules are stacked in an almost parallel fashion due to the presence of π···π interaction between the aromatic systems. Indeed, the mean planes passing through the anthracene moieties of cations A and B form an angle of 3.3° with a distance of 3.50 Å between the planes. A further and distinct network of AEPy^2+^ cations is established between an AB unit interacting through C–H···π bonds with a further AEPy^2+^ cation and extending along the *a*-direction (Figure S6). While the AEPy^2+^ cations inside the AB unit interact through π···π bonds as previously described, the C–H···π bonds involve the methyl group bound to the pyridinium nitrogen, which interacts with the pyridinium (Py) aromatic system of neighboring AEPy^2+^ cation. The distance between the centroid of the pyridinium ring and C59 is 4.11 Å, while the C–H···Py distance is 3.66 Å.

**Figure 5:**
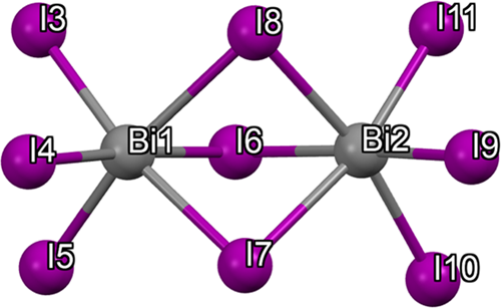
Bi_2_I_9_^3–^ polyhedron. The two face-sharing BiI_6_^3–^ octahedra units are highly distorted due to stereochemically active 6s lone pair.

Similarly to the case of (CH_3_NH_3_)_3_Bi_2_I_9_, the face-sharing BiI_6_^3–^ octahedra in the Bi_2_I_9_^3–^ units (Figure 5) are highly distorted with uneven interatomic distances and bond angles, thus suggesting a stereochemically active 6s lone pair, similar to the case of Pb-based HMHPs.^30^ This distortion agrees with those observed in other 0D bismuth iodide perovskites,^19,20^ i.e., the Bi–I distances in Bi1–I3, Bi1–I4, Bi1–I5, Bi2–I9, Bi2–I10, and Bi2–I11 are sensibly shorter than the distances Bi1–I6, Bi1–I7, Bi1–I8, Bi2–I6, Bi2–I7, and Bi2–I8 (Table S1 of the Supporting Information). Also, the bond angles show the same behavior seen for other perovskites, with the I–Bi–I angles formed with I atoms shared by the two octahedra having values below 90° versus values over 90° for I–Bi–I angles formed with no-shared I atoms (Table S2 of the Supporting Information).

The Bi_2_I_9_^3–^ anions interact with the cations via the following hydrogen bonds: I3-H36C (3.07 Å), I7-H36A (2.99 Å), I3-H57 (3.08 Å), I10-H31 (3.10 Å), and I5-H23 (3.15 Å). As in the case of phenylviologen lead iodide, these interactions suggest the donor–acceptor semiconductor behavior of AEPyBiI with charge transfer processes from Bi_2_I_9_^3–^ anions to the cations.^22^

The bond lengths in the cation are consistent with those expected. In particular, the C–C distances in the acetylenic linkers (1.19 Å) between the pyridinic rings and the anthracene unit are consistent with the expected value for a triple bond (1.189 Å).^31^

**Figure 6:**
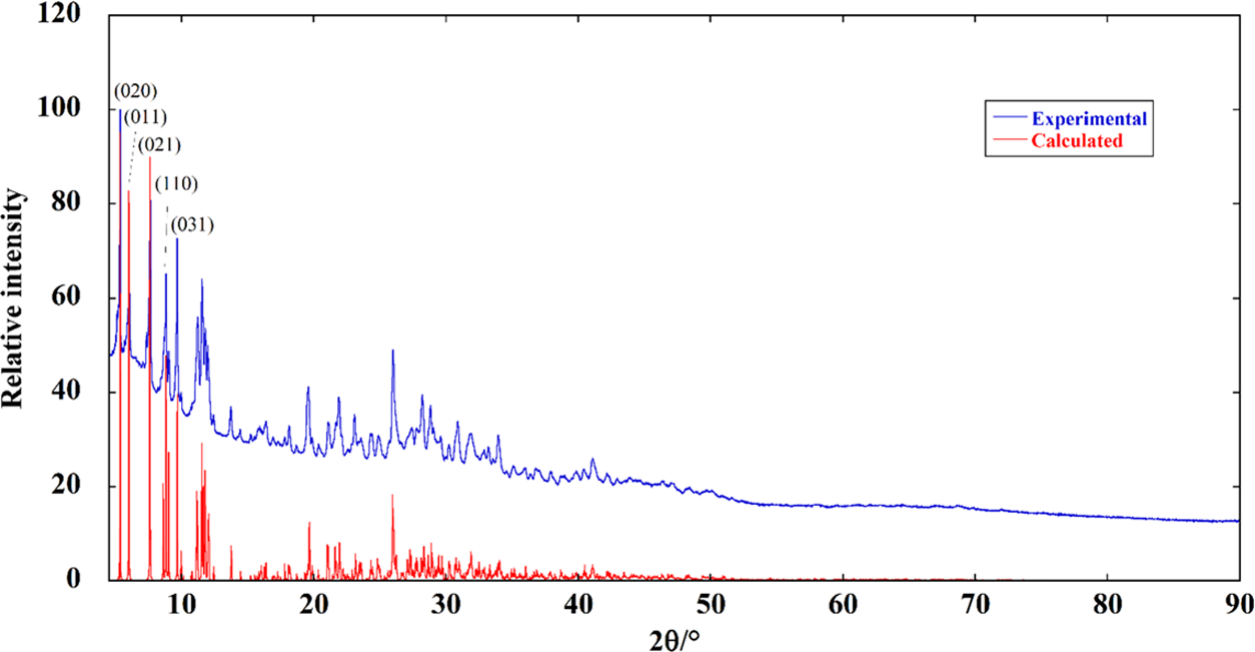
Experimental versus calculated powder diffraction pattern of AEPyBiI.

The powder diffraction pattern of AEPyBiI is shown in Figure 6 together with the pattern calculated from the solved crystal structure. The five most intense reflections are indexed. The agreement is excellent. The agreement between the powder diffraction pattern and the structure obtained by the single-crystal data is further confirmed by the Le Bail fit (Figure S7 of the Supporting Information, *R*_p_ = 0.338, *R*_wp_ = 0.696, *R*_Bragg_ = 3.383).

The composition of AEPyBiI was investigated using CHNS analysis and ESI mass spectrometry. The CHNS analysis of AEPyBiI gave the following results: C 24.94%, H 1.65%, and N 2.40%. The calculated values are C 24.84%, H 1.53%, and N 1.93% for (C_30_H_22_N_2_)_3_Bi_4_I_18_. The slight discrepancy between experimental and calculated values has to be attributed to the entrapment of DMF molecules in the crystal structure during crystal growth. In fact, during the structure solution procedure, disordered DMF molecules were found. The composition of AEPyBiI has been also verified by ESI-MS spectra (Figure S8 in the Supporting Information), where the dominant peaks in the positive-ion spectrum are due to the intact divalent cation (*m*/*z* = 205) and the cation loses a methyl group and is consequently monovalent (*m*/*z* = 395).

**Figure 7:**
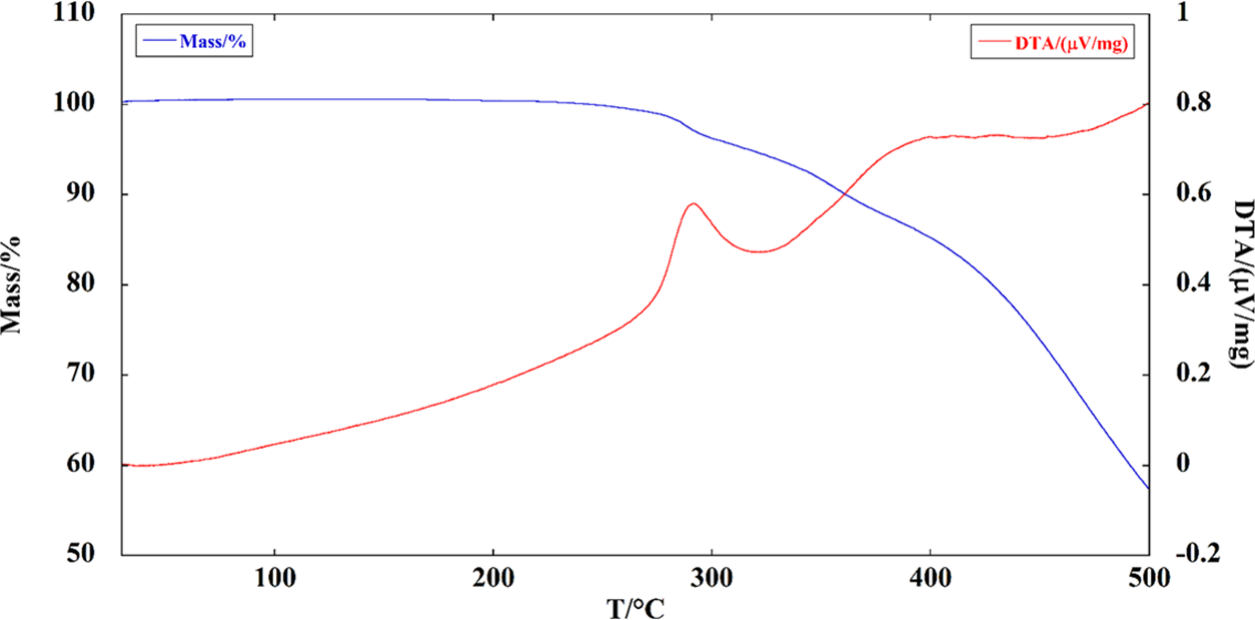
TG-DTA of AEPyBiI.

The thermal stability of AEPyBiI was evaluated by TG-DTA (Figure 7).

The material does not show thermal events up to approximately 300 °C and then starts losing weight. Then, two very broad endothermic events appear in the DTA profile, the first one starting around 300 °C and the second around 400 °C, both marked by a change in the slope of the TG curve, thus representing different stages of the material decomposition process. The fist endothermic peak can be attributed to structural solvent loss and degradation of the organic cation, while the second one, associated with a higher mass loss rate and constant slope, is assigned to the evaporation of BiI_3_, which possesses a very high vapor pressure above 300 °C (1 atm at 316 °C).^32^ No DTA peaks attributable to phase transitions are present in the DTA curve. AEPyBiI consequently possesses a remarkable thermal stability, and its crystal structure remains constant up to the decomposition temperature.

To test the stability of AEPyBiI toward water at room temperature, a powder sample was immersed in distilled water for 1 h, filtered, and dried. An X-ray powder diffraction scan was performed on the water-treated sample. The background-subtracted pattern (Figure S9 in the Supporting Information) does not show significant differences with the as-prepared sample (minor differences in relative intensities are due to preferred orientation effects that are unavoidable in a static Bragg–Brentano geometry like the one used here), thus demonstrating its water stability at room temperature.

All these factors are positive in the perspective of the use of AEPyBiI in real devices.

### Spectroscopic Characterization

**Figure 8:**
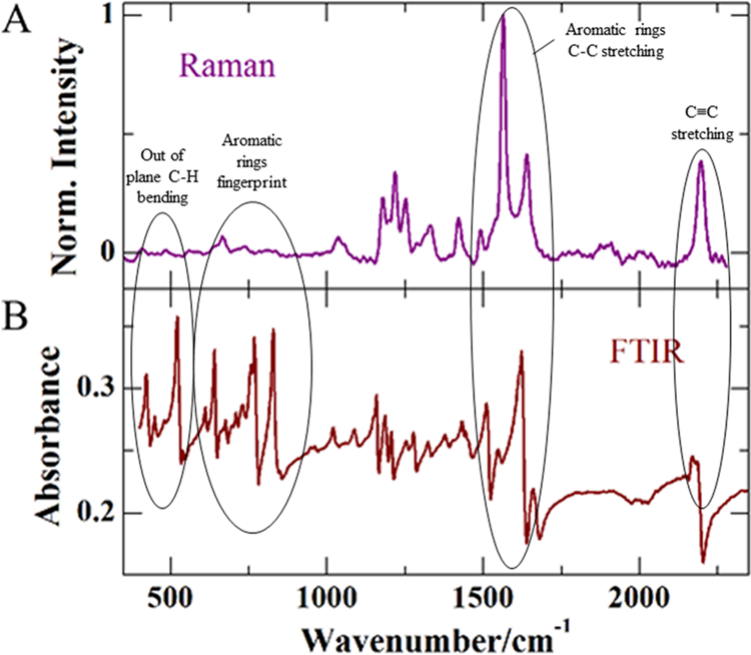
Raman (A) and FTIR (B) spectra of AEPyBiI in the range from 350 to 2350 cm^–1^.

AEPyBiI was also characterized by Raman and Fourier transform infrared (FTIR) spectroscopies, as shown in Figure 8. The two techniques provide consistent results, showing an overall good agreement between the various spectral features observed.

The bands are due only to the organic cation, considering the fact that Bi and I are heavy atoms and consequently the vibrations due to the inorganic sublattice cannot be seen in the instrument’s spectral range (400–4000 cm^–1^). The peak at 2165 cm^–1^ can be assigned to the carbon–carbon stretching of the two triple bonds present in the molecule, while the intense FTIR features in the range 900–650 cm^–1^ are typical of aromatic structures, as in our case (the same features are observed also in the Raman spectrum, yet less pronounced). The intense peaks around 1600 cm^–1^ can be assigned to the carbon–carbon stretching in aromatic rings. The 400–650 cm^–1^ region show peaks that can be attributed to the out-of-plane C–H bending vibrations.^33^ Both Raman and FTIR measurements were taken also in the range from 2350 to 4000 cm^–1^, but no peaks were observed.

**Figure 9:**
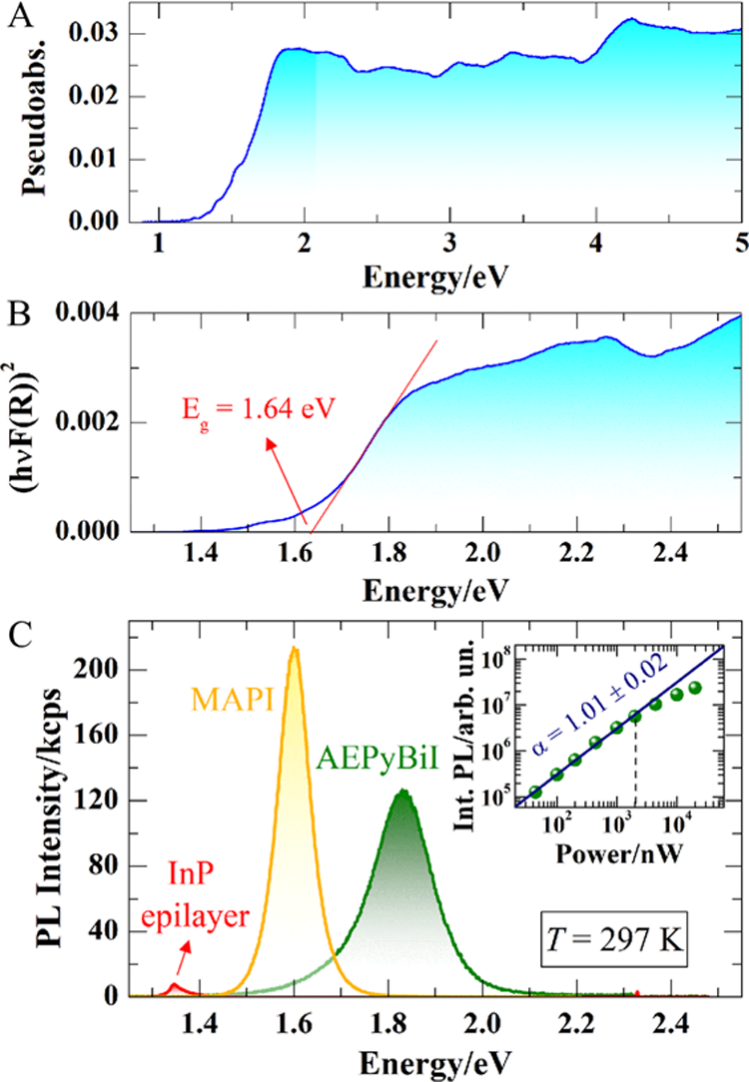
(A) UV–vis spectrum of AEPyBiI. (B) Tauc’s plot (assuming a direct optical transition) for the determination of the band gap value *E*_g_. (C) photoluminescence (PL) spectrum of AEPyBiI as compared to the PL spectrum of a high-quality InP epilayer and of MAPI. The excitation power was *P* = 2 μW. The inset shows the integrated intensity of the PL signal of AEPyBiI as a function of the excitation laser power. The data were fitted up to the dashed line (2 μW), leading to a linear behavior with exponent α shown in the plot.

The optical properties of AEPyBiI were probed by diffuse reflectance UV–vis spectroscopy and by photoluminescence (PL) spectroscopy. The pseudoabsorbance spectrum, as determined by UV–vis measurements, is shown in Figure 9A. The corresponding Tauc’s plot,^34^ evaluated by assuming a direct optical transition, can be seen in panel B. From the Tauc’s plot, we estimate an optical band gap energy *E*_g_ of 1.64 eV.

To probe the optical efficiency of AEPyBiI and its potentiality for optoelectronic applications, we performed PL measurements at room temperature. The sample was excited by a 532 nm laser via a 100× objective; the signal was collected through the same objective, spectrally dispersed through a 150 grooves/nm monochromator, and detected with a Si-CCD. The spectrum of the perovskite can be seen in Figure 9C. The PL band is centered at about 1.83 eV, and thus about 200 meV above the gap estimated by the Tauc’s plot. For the evaluation of the efficiency of the emission of AEPyBiI, the figure also shows the spectrum of a high-quality 3-micron-thick (100) InP epilayer grown by metal–organic chemical vapor deposition at a temperature of 650 °C, a highly emissive material whose emission properties are well established, taken exactly under the same experimental conditions of the perovskite. AEPyBiI is indeed much more intense, its integrated intensity being larger than that of the InP signal by a factor of ∼35. We also compared the PL signal of AEPyBiI to that of methylammonium lead iodide CH_3_NH_3_PbI_3_ (MAPI), whose spectrum is shown in the figure, and found that the two are characterized by a comparable PL efficiency (the integrated intensity of MAPI being a factor of 1.1 larger than that of AEPyBiI). AEPyBiI features a very homogeneous PL signal (see Supporting Information, Figure S10). To test its robustness upon photoexcitation, we performed PL measurements by varying the excitation power from 40 nW to 20 μW. In this range, the lineshape of the PL signal does not show significant variation (see Supporting Information, Figure S11). The integrated area of the PL signal increases linearly up to about 2 μW, as can be seen in the inset of Figure 9C, where the data were fitted through the equation *I* = A*·P*^α^, where *I* is the integrated intensity, *P* is the laser power, A is a scaling constant, and α is the exponent, which was found to be equal to 1.01 ± 0.02 by the fit, suggesting a modest density of nonradiative recombination channels. For *P* > 5 μW, the intensity deviates from the linear behavior, suggesting that a degradation of the sample is starting to occur.

## Conclusions

A novel 0D hybrid bismuth iodide perovskite with a highly conjugated quaternary ammonium cation has been synthesized by a very simple procedure mixing solutions of bismuth and organic cation iodides in acetone and water, respectively, at room temperature. The so-obtained perovskite was characterized structurally, chemically, and optically. It shows remarkable chemical and thermal stability, being insensitive to water and decomposing around 300 °C. The material is highly photoluminescent, emitting in the red region of the visible spectrum with an integrated emission intensity in excess of a factor of 30 larger than InP and comparable to MAPI in the same excitation conditions. The emissive properties of the material, coupled with the easy synthesis and the lack of toxicity of bismuth, make it interesting for optoelectronic applications such as light-emitting devices.

## Experimental section

The synthesis of 4,4′-(anthracene-9,10-diylbis(ethyne-2,1-diyl))bis(1-methyl-1-pyridinium) iodide is described in the Supporting Information. MAPI was prepared according to the literature procedure.^10^

### Single-Crystal Structure Analysis

X-ray diffraction measurements were performed at the XRD1 beamline of the Elettra synchrotron radiation facility (Trieste, Italy)^35,36^ at room temperature (293 K). X-ray wavelength was set to 0.7 Å, while a Pilatus 2M (Dectris) detector was used for the diffraction experiments. Unit cell assignment, integration, and data reduction were performed with the XDS program;^37^ empirical absorption correction was applied using SADABS software.^38^ The structure was solved using SHELXT,^39^ while Fourier analysis and refinement were performed by the full-matrix least-squares methods based on *F*^2^ implemented in SHELXL^40^ through the OLEX2 software.^41^ Anisotropic thermal motion refinement have been used for all atoms with full occupancy with hydrogen atoms included at calculated positions with isotropic *U* factors = 1.2·Ueq (Ueq being the equivalent isotropic thermal factor of the bonded nonhydrogen atom). According to a difference Fourier map, a disordered DMF molecule was located and modeled. The disordered DMF molecule was then refined with full occupancy but keeping both geometry and isotropic thermal parameters restrained.

### Powder X-ray Diffraction

Powder diffraction analysis was performed with a Malvern Panalytical X’Pert Pro MPD diffractometer (Cu Kα radiation, λ = 1.54184 Å). The diffractometer is equipped with a ultrafast X’Celerator RTMS detector. The used angular resolution in 2θ is 0.001°. The scans were collected in the 4.5–90° 2θ angular range. A beam knife was used in the 4.5–6.5° range to reduce the background. The Le Bail fit of the powder diffraction pattern was performed using the EXPO 2014 software.^42^

### ESI-MS

The ESI-MS analysis (positive ions) was performed with a Thermo Scientific, TSQ QUANTUM ACCESS spectrometer equipped with a triple quadrupole analyzer. The compound was dissolved in methanol, in which it was slightly soluble.

### CHNS Analysis

The C, H, N, and S contents of the material were determined with an EA 1110 CHNS-O elemental analyzer.

### Thermogravimetry-Differential Thermal Analysis (TG-DTA)

TG-DTA was performed using a Netzsch STA 409 PC Luxx simultaneous thermal analyzer. The analysis was performed under flowing Ar atmosphere (85 cm^3^/min @ STP, purity ≥99.9995%) in an alumina crucible. A thermal scan rate of 10 K/min in the 30–500 °C temperature range was used.

### UV–vis Spectroscopy

The diffuse reflectance UV–vis spectrum was acquired with a Shimadzu (Japan) UV2600 UV–vis spectrophotometer. The spectrophotometer is equipped with an ISR-2600 Plus integrating sphere (BaSO_4_ reference).

### FTIR Spectroscopy

FTIR spectrum was acquired with a Bruker Alpha spectrophotometer in the 400–4000 cm^–1^ wavenumber range (resolution 4 cm^–1^). Data were acquired in ATR mode using the ATR Platinum Diamond 1 accessory.

### Raman Spectroscopy

For Raman measurements, the excitation laser was provided by a single-frequency Nd:YVO_4_ laser (DPSS series by Lasos) emitting at 532 nm. The Raman signal was spectrally dispersed by a ACTON SP750 monochromator with a focal length of 750 mm and equipped with a 300 grooves/mm grating. The signal was detected by a back-illuminated N_2_-cooled Si-CCD camera (100BRX by Princeton Instruments). The laser light was filtered out by a very sharp long-pass Razor edge filter (Semrock). The spectral resolution was 2.8 cm^–1^. A 100× objective with NA = 0.9 was employed to excite and collect the light in a backscattering configuration.

### PL Spectroscopy

PL measurements were taken in the same experimental configuration used for Raman measurements. In this case, the signal was spectrally dispersed by a Princeton Isoplane160 monochromator with a focal length of 200 mm and equipped with a 150 grooves/mm grating. To have reliable information of the PL lineshape and intensity, the system response was duly taken into account. The system response was measured using a blackbody source and comparing the measured spectrum with the blackbody nominal one.

## Supporting Information Available

Fluorescence spectrum of of AEPy^2+^ in water (Figure S1); Photo of a fluorescent water solution of AEPy^2+^ (Figure S2); AEPyBiI stick-and-ball representation together with the labelling scheme (Figure S3); AEPyBiI including the symmetry generated half AEPy^2+^ colored in orange (Figure S4); stick-and-ball representation of the (ABA)(ABA) network (Figure S5); stick-and-ball representation of the (AB)··· AEPy^2+^ interaction network (Figure S6); Le Bail fit of the experimental powder diffraction pattern (Figure S7); positive ions ESI mass spectrum (Figure S8); Background-subtracted XRD powder patterns (Figure S9); PL spectra of AEPyBiI acquired on 7 different points of the powder (Figure S10); normalized PL spectra (Figure S11); Bismuth-iodine interatomic distances (Table S1); I-Bi-I angles (Table S2) (PDF)

## References

[ref1] Green M. A.; Dunlop E. D.; Hohl-Ebinger J.; Yoshita M.; Kopidakis N.; Hao X. Solar cell efficiency tables (version 59). Prog. Photovolt.: Res. Appl. 2022, 30, 3–12. 10.1002/pip.3506.

[ref2] Suresh Kumar N.; Chandra Babu Naidu K. A review on perovskite solar cells (PSCs), materials and applications. J. Mater. 2021, 7, 940–956. 10.1016/j.jmat.2021.04.002.

[ref3] Huang J.; Yuan Y.; Shao Y.; Yan Y. Understanding the physical properties of hybrid perovskites for photovoltaic applications. Nat. Rev. Mater. 2017, 2, 17042 10.1038/natrevmats.2017.42.

[ref4] Younis A.; Lin C.; Guan X.; Shahrokhi S.; Huang C.; Wang Y.; He T.; Singh S.; Hu L.; Duran Retamal J. R.; He J.; Wu T. Adv. Mater. 2021, 33, 2005000 10.1002/adma.202005000. 33938612

[ref5] Wang Y.; Liu Y.; Cao S.; Wang J. A review on solution-processed perovskite/organic hybrid photodetectors. J. Mater. Chem. C 2021, 9, 5302–5322. 10.1039/D1TC00643F.

[ref6] Veldhuis S. A.; Boix P. P.; Yantara N.; Li M.; Chien Sum V.; Mathews N.; Mhaisalkar S. G. Perovskite Materials for Light-Emitting Diodes and Lasers. Adv. Mater. 2016, 28, 6804–6834. 10.1002/adma.201600669. 27214091

[ref7] Stylianakis M. M.; Maksudov T.; Panagiotopoulos A.; Kakavelakis G.; Petridis K. Inorganic and Hybrid Perovskite Based Laser Devices: A Review. Materials 2019, 12, 859 10.3390/ma12060859. 30875786 PMC6470628

[ref8] Zhou C.; Lin H.; Lee S.; Chaaban M.; Ma B. Organic–inorganic metal halide hybrids beyond perovskites. Mater. Res. Lett. 2018, 6, 552–569. 10.1080/21663831.2018.1500951.

[ref9] Schmidt-Mende L.; Dyakonov V.; Olthof S.; Ünlü F.; Moritz Trong Lê K.; Mathur S.; Karabanov A. D.; Lupascu D. C.; Herz L. M.; Hinderhofer A.; et al. Roadmap on organic–inorganic hybrid perovskite semiconductors and devices. APL Mater. 2021, 9, 109202 10.1063/5.0047616.

[ref10] Brunetti B.; Cavallo C.; Ciccioli A.; Gigli G.; Latini A. On the Thermal and Thermodynamic (In)Stability of Methylammonium Lead Halide Perovskites. Sci. Rep. 2016, 6, 31896 10.1038/srep31896. 27545661 PMC4992962

[ref11] Latini A.; Gigli G.; Ciccioli A. A study on the nature of the thermal decomposition of methylammonium lead iodide perovskite, CH_3_NH_3_PbI_3_: an attempt to rationalise contradictory experimental results. Sustainable Energy Fuels 2017, 1, 1351–1357. 10.1039/C7SE00114B.

[ref12] Ciccioli A.; Latini A. Thermodynamics and the Intrinsic Stability of Lead Halide Perovskites CH_3_NH_3_PbX_3_. J. Phys. Chem. Lett. 2018, 9, 3756–3765. 10.1021/acs.jpclett.8b00463. 29901394

[ref13] Panetta R.; Righini G.; Colapietro M.; Barba L.; Tedeschi D.; Polimeni A.; Ciccioli A.; Latini A. Azetidinium lead iodide: synthesis, structural and physico-chemical characterization. J. Mater. Chem. A 2018, 6, 10135–10148. 10.1039/C8TA02210K.

[ref14] Ciccioli A.; Panetta R.; Luongo A.; Brunetti B.; Vecchio Ciprioti S.; Mele M. L.; Latini A. Stabilizing lead halide perovskites with quaternary ammonium cations: the case of tetramethylammonium lead iodide. Phys. Chem. Chem. Phys. 2019, 21, 24768–24777. 10.1039/C9CP04051J. 31686067

[ref15] Park C.; Choi J.; Min J.; Cho K. Suppression of Oxidative Degradation of Tin-Lead Hybrid Organometal Halide Perovskite Solar Cells by Ag Doping. ACS Energy Lett. 2020, 5, 3285–3294. 10.1021/acsenergylett.0c01648.

[ref16] Adjogri S. J.; Meyer E. L. A Review on Lead-Free Hybrid Halide Perovskites as Light Absorbers for Photovoltaic Applications Based on Their Structural, Optical, and Morphological Properties. Molecules 2020, 25, 5039 10.3390/molecules25215039. 33143007 PMC7662694

[ref17] Zhang L.; Wang K.; Zou B. Bismuth Halide Perovskite-Like Materials: Current Opportunities and Challenges. ChemSusChem 2019, 12, 1612–1630. 10.1002/cssc.201802930. 30693678

[ref18] Lou Y.; Fang M.; Chen J.; Zhao Y. Formation of highly luminescent cesium bismuth halide perovskite quantum dots tuned by anion exchange. Chem. Commun. 2018, 54, 3779–3782. 10.1039/C8CC01110A. 29594292

[ref19] Yang B.; Chen J.; Hong F.; Mao X.; Zheng K.; Yang S.; Li Y.; Pullerits T.; Deng W.; Han K. Lead-Free, Air-Stable All-Inorganic Cesium Bismuth Halide Perovskite Nanocrystals. Angew. Chem., Int. Ed. 2017, 56, 12471–12475. 10.1002/anie.201704739. 28796440

[ref20] Hoye R. L. Z.; Brandt R. E.; Osherov A.; Stevanovic V.; Stranks S. D.; Wilson M. W. B.; Kim H.; Akey A. J.; Perkins J. D.; Kurchin R. C.; et al. Methylammonium Bismuth Iodide as a Lead-Free, Stable Hybrid Organic-Inorganic Solar Absorber. Chem. – Eur. J. 2016, 22, 2605–2610. 10.1002/chem.201505055. 26866821

[ref21] Tang Z.; Guloy A. M. A Methylviologen Lead(II) Iodide: Novel [PbI_3_^-^]_∞_ Chains with Mixed Octahedral and Trigonal Prismatic Coordination. J. Am. Chem. Soc. 1999, 121, 452–453. 10.1021/ja982702i.

[ref22] Latini A.; Quaranta S.; Menchini F.; Lisi N.; Di Girolamo D.; Tarquini O.; Colapietro M.; Barba L.; Demitri N.; Cassetta A. A novel water-resistant and thermally stable black lead halide perovskite, phenyl viologen lead iodide C_22_H_18_N_2_(PbI_3_)_2_. Dalton Trans. 2020, 49, 2616–2627. 10.1039/C9DT04148F. 32039432

[ref23] Blundo E.; Polimeni A.; Meggiolaro D.; D’Annibale A.; Romagnoli L.; Felici M.; Latini A. Brightly Luminescent and Moisture Tolerant Phenyl Viologen Lead Iodide Perovskites for Light Emission Applications. J. Phys. Chem. Lett. 2021, 12, 5456–5462. 10.1021/acs.jpclett.1c01271. 34081469 PMC8280716

[ref24] Yue C.-Y.; Zhao H.; Jiang H.; Guo Y.; Che H.; Li J.; Chu W.; Yuan Y.; Jing Z.; Lei X. Large Conjugated Organic Cations Sensitized Hybrid Lead Halides as Visible Light Driven Photocatalysts. Cryst. Growth Des. 2019, 19, 4564–4570. 10.1021/acs.cgd.9b00395.

[ref25] Wei Q.; Ge B.; Zhang J.; Sun A.; Li J.; Han S.; Wang G. Tripyridine-Derivative-Derived Semiconducting Iodo-Argentate/Cuprate Hybrids with Excellent Visible-Light-Induced Photocatalytic Performance. Chem. Asian J. 2019, 14, 269–277. 10.1002/asia.201801555. 30521150

[ref26] Li F.-Y.; Wen X.; Xue Z.; Pan J.; Wei Q.; Wei L. Large Conjugated Bis/Triimidazolium Derivatives Directed Iodobismuthates(III): Syntheses, Structures, and Visible-Light-Induced Photocatalytic Properties. Cryst. Growth Des. 2022, 22, 4601–4609. 10.1021/acs.cgd.2c00521.

[ref27] Sonogashira K. Development of Pd–Cu catalyzed cross-coupling of terminal acetylenes with sp^2^-carbon halides. J. Organomet. Chem. 2002, 653, 46–49. 10.1016/S0022-328X(02)01158-0.

[ref28] Eckhardt K.; Bon V.; Getzschmann J.; Grothe J.; Wisser F. M.; Kaskel S. Crystallographic insights into (CH_3_NH_3_)_3_(Bi_2_I_9_): a new lead-free hybrid organic–inorganic material as a potential absorber for photovoltaics. Chem. Commun. 2016, 52, 3058–3060. 10.1039/C5CC10455F. 26810737

[ref29] Park B.-W.; Philippe B.; Zhang X.; Rensmo H.; Boschloo G.; Johansson E. M. J. Bismuth Based Hybrid Perovskites A_3_Bi_2_I_9_ (A: Methylammonium or Cesium) for Solar Cell Application. Adv. Mater. 2015, 27, 6806–6813. 10.1002/adma.201501978. 26418187

[ref30] D’Annibale A.; Panetta R.; Tarquini O.; Colapietro M.; Quaranta S.; Cassetta A.; Barba L.; Chita G.; Latini A. Synthesis, physico-chemical characterization and structure of the elusive hydroxylammonium lead iodide perovskite NH_3_OHPbI_3_. Dalton Trans. 2019, 48, 5397–5407. 10.1039/C9DT00690G. 30946403

[ref31] Prince E. International Tables for Crystallography. Volume C, Mathematical, Physical and Chemical Tables.; Kluwer: The Netherlands, 2004.

[ref32] Chen X.; Myung Y.; Thind A.; Gao Z.; Yin B.; Shen M.; Cho S. B.; Cheng P.; Sadtler B.; Mishra R.; Banerjee P. Atmospheric pressure chemical vapor deposition of methylammonium bismuth iodide thin films. J. Mater. Chem. A 2017, 5, 24728–24739. 10.1039/C7TA06578G.

[ref33] Silverstein R. M.; Webster F. X.; Kiemle D. J. Spectrometric Identification of Organic Compounds, Wiley: Hoboken, USA, 2005.

[ref34] Tauc J.; Grigorovici R.; Vancu A. Optical Properties and Electronic Structure of Amorphous Germanium. Phys. Status Solidi B 1966, 15, 627–637. 10.1002/pssb.19660150224.

[ref35] Bernstorff S.; Busetto E.; Gramaccioni C.; Lausi A.; Olivi L.; Zanini F.; Savoia A.; Colapietro M.; Portalone G.; Camalli M.; et al. The macromolecular crystallography beamline at ELETTRA. Rev. Sci. Instrum. 1995, 66, 1661–1664. 10.1063/1.1145875.

[ref36] Lausi A.; Polentarutti M.; Onesti S.; Plaisier J. R.; Busetto E.; Bais G.; Barba L.; Cassetta A.; Campi G.; Lamba D.; et al. Status of the crystallography beamlines at Elettra. Eur. Phys. J. Plus 2015, 130, 43 10.1140/epjp/i2015-15043-3.

[ref37] Kabsch W. XDS. Acta Crystallogr. D 2010, 66, 125–132. 10.1107/S0907444909047337. 20124692 PMC2815665

[ref38] Sheldrick G. M. SADABS, Software for Empirical Absorption Corrections; University of Göttingen: Göttingen, 1996.

[ref39] Sheldrick G. M. SHELXT - Integrated space-group and crystal-structure determination. Acta Crystallogr. A 2015, 71, 3–8. 10.1107/S2053273314026370. PMC4283466 25537383

[ref40] Sheldrick G. M. Crystal structure refinement with SHELXL. Acta Crystallogr. C 2015, 71, 3–8. 10.1107/S2053229614024218. PMC4294323 25567568

[ref41] Dolomanov O. V.; Bourhis L. J.; Gildea R. J.; Howard J. A. K.; Puschmann H. OLEX2: a complete structure solution, refinement and analysis program. J. Appl. Crystallogr. 2009, 42, 339–341. 10.1107/S0021889808042726.

[ref42] Altomare A.; Cuocci C.; Giacovazzo C.; Moliterni A.; Rizzi R.; Corriero N.; Falcicchio A. EXPO2013: a kit of tools for phasing crystal structures from powder data. J. Appl.Crystallogr. 2013, 46, 1231–1235. 10.1107/S0021889813013113.

