# Supplementary material for: Synthesis, Structure, and Characterization of 4,4′-(Anthracene-9,10-diylbis(ethyne-2,1-diyl))bis(1-methyl-1-pyridinium) Bismuth Iodide (C30H22N2)3Bi4I18, an Air, Water, and Thermally Stable 0D Hybrid Perovskite with High Photoluminescence Efficiency
Source: Cryst Growth Des. 2022 Nov 22;22(12):7426–33. doi: 10.1021/acs.cgd.2c01005 (PMC9732820; doi:10.1021/acs.cgd.2c01005)
Supplement: Supplementary file 1 — cg2c01005_si_001.pdf [file cg2c01005_si_001.pdf]

Supporting Information

Synthesis, structure and characterization of 4,4'-(anthracene-9,10-diylbis(ethyne-2,1-diyl))bis(1-methyl-1-pyridinium) bismuth iodide $(C_{30}H_{22}N_2)_3Bi_4I_{18}$, an air, water and thermally stable 0D hybrid perovskite with high photoluminescence efficiency

Lorenza Romagnoli[1], Andrea D'Annibale[1], Elena Blundo[2*], Antonio Polimeni[2], Alberto Cassetta[3*], Giuseppe Chita[3], Riccardo Panetta[4], Andrea Ciccioli[1], Alessandro Latini[1*]

[1] *Dipartimento di Chimica, Sapienza Università di Roma, Piazzale Aldo Moro 5, 00185 Roma, Italy*

[2] *Dipartimento di Fisica, Sapienza Università di Roma, Piazzale Aldo Moro 5, 00185 Roma, Italy*

[3] *Consiglio Nazionale delle Ricerche - Istituto di Cristallografia, Sede Secondaria di Trieste, Area Science Park – Basovizza, Strada Statale 14, km 163.5, 34149 Trieste, Italy*

[4] *Ispa - Istituto Sperimentale Problematiche Ambientali, Via San Nicandro snc - 03042 Atina (FR), Italy*

*corresponding authors

Synthesis of 4,4'-(anthracene-9,10-diylbis(ethyne-2,1-diyl))bis(1-methyl-1-pyridinium) iodide ($AEPyI_2$)

All the chemicals used were purchased from Merck and used as received unless otherwise stated.

Synthesis of 4-((trimethylsilyl)ethynyl)pyridine:[1]

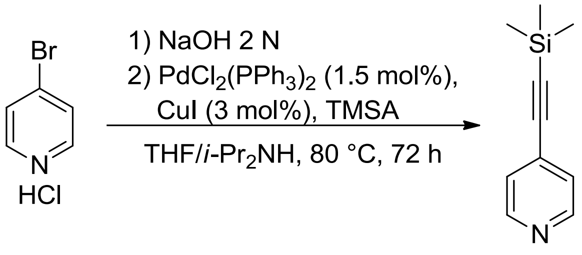

1 (60%)

In a flame-dried Schlenk tube, under argon atmosphere, 60 mL of a degassed 3:1 THF/i-Pr_2NH mixture and 7.3 g (37.5 mmol) of 4-bromopyridine hydrochloride (previously washed with a 2 N aqueous NaOH solution and extracted three times with CH_2Cl_2) are introduced. Then, 400 mg (0.57 mmol) of $Pd(PPh_3)_2Cl_2$, 195 mg (1.0 mmol) of CuI and 7.0 mL (50.5 mmol) of trimethylsilylacetylene are added and the mixture is heated to 80 °C and stirred for 3 days. After that, the reaction is cooled to room temperature, the resulting mixture is filtered to remove the solids and washed with CH_2Cl_2, then extracted with H_2O (100 mL), dried over anhydrous Na_2SO_4 and concentrated under vacuum. The crude product is purified with column chromatography (9:1 petroleum ether/ethyl acetate) to yield 3.9 g of pure **1** as a yellow-brownish oil (60%). ^{1}H-NMR ($CDCl_3$, 400 MHz) δ 8.55 (m, 2H), 7.30 (m, 2H) 0.25 (s, 9H); ^{13}C-NMR ($CDCl_3$, 100 MHz) δ 149.65, 131.26, 125.84, 101.94, 100.00, -0.32.

Synthesis of 4-ethynylpyridine:[1]

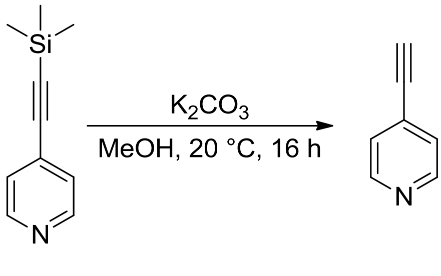

1 **2**

In a round-bottom flask, 3.9 g (22.3 mmol) of 4-((trimethylsilyl)ethynyl)pyridine are dissolved in 55 mL of methanol and 3.48 g (25.2 mmol) of K_2CO_3 are added. The resulting suspension is stirred overnight at room temperature, protected from light, then 50 mL of H_2O are added, and the solution is extracted with CH_2Cl_2 (3x50 mL). The combined organic phases are dried over anhydrous Na_2SO_4 and concentrated under vacuum at room temperature, to yield 2.9 g of crude **2** as a light-sensitive and volatile white solid, which is used without further purification. ^{1}H-NMR ($CDCl_3$, 400 MHz) δ 8.59 (m, 2H), 7.34 (m, 2H) 3.29 (s, 1H); ^{13}C-NMR ($CDCl_3$, 100 MHz) δ 149.81, 130.33, 126.09, 81.87, 80.94.

Synthesis of 9,10-bis(pyridin-4-ylethynyl)anthracene:[2]

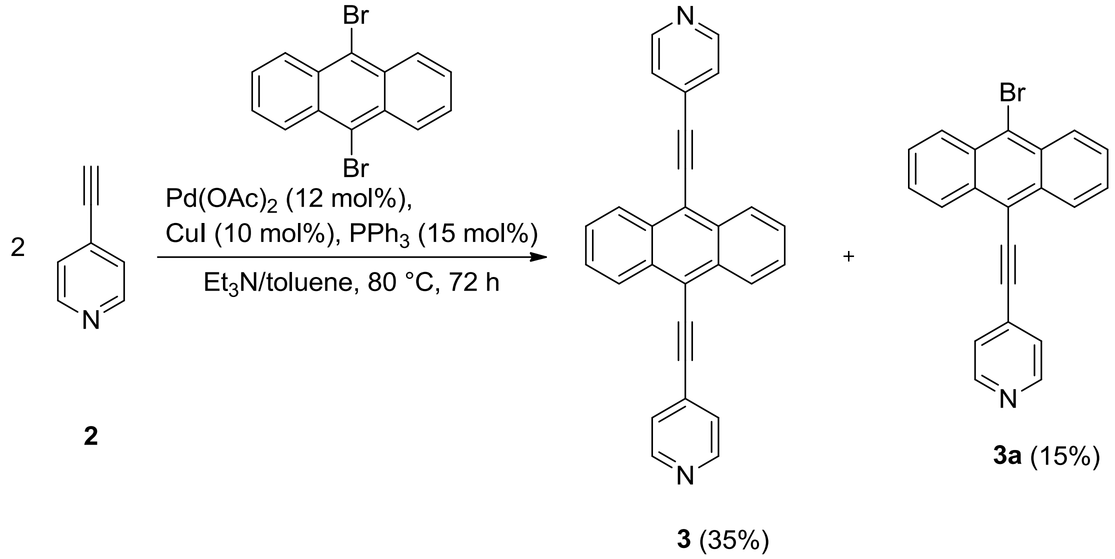

In a flame-dried Schlenk tube, under argon atmosphere, 25 mL of a degassed 1:1 toluene/Et_3N mixture is introduced, together with 400 mg (1.2 mmol) of dibromoanthracene, 27 mg (0.14 mmol) of CuI, 27 mg (0.12 mmol) of $Pd(OAc)_2$ and 54 mg (0.21 mmol) of PPh_3, and the mixture is stirred at room temperature; after that, 375 mg (3.6 mmol) of 4-ethynylpyridine are added and the temperature is increased to 80 °C. The reaction mixture is stirred for 3 days, then it is cooled to room temperature and filtered to remove the solids. The solvent is then evaporated under reduced pressure and the crude is separated by column chromatography (7:3 petroleum ether/ethyl acetate → ethyl acetate) to yield 158 mg of compound **3** (35%) along with 54 mg of by-product **3a** (15%). ^{1}H-NMR ($CDCl_3$, 400 MHz) δ 8.73 (m, 4H), 8.65 (m, 4H), 7.70 (m, 4H), 7.63 (m, 4H); ^{13}C-NMR ($CDCl_3$, 100 MHz) δ 149.92, 132.58, 131.67, 127.47, 127.02, 125.53, 118.51, 99.68, 90.74.

Synthesis of 4,4'-(anthracene-9,10-diylbis(ethyne-2,1-diyl))bis(1-methyl-1-pyridinium) iodide (**AEPyI2**):[3]

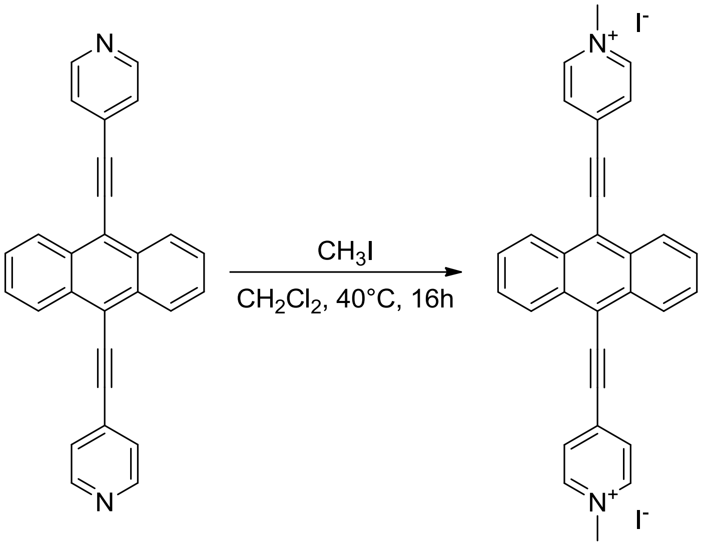

3 **AEPyI2** (72%)

In a flame-dried Schlenk tube, 146 mg (0.38 mmol) of **3** are dissolved in anhydrous CH_2Cl_2 and 1.3 mL (20.9 mmol) of CH_3I are added under argon atmosphere. The mixture is stirred at 40 °C overnight, then it is cooled to room temperature and filtered under vacuum; the collected solid is washed with portions of CH_2Cl_2 and dried for 1 hour under suction, to yield 184 mg (0.28 mmol) of **AEPyI2** as a dark red solid (72%). ^{1}H-NMR (DMSO-d_6, 400 MHz) δ 9.13 (m, 4H), 8.84 (m, 4H), 8.67 (m, 4H), 7.92 (m, 4H), 4.38 (s, 6H); ^{13}C-NMR (DMSO-d_6, 100 MHz) δ 146.14, 138.12, 132.59, 129.76, 129.61, 127.46, 118.19, 99.39, 97.61, 48.36. MS (+ESI): *m/z* 205.5 [M^{2+}].

References:

1. Ziessel, R.; Suffert, J.; Youinou, M.T. General Method for the Preparation of Alkyne-Functionalized Oligopyridine Building Blocks. *J. Org. Chem.* **1996**, 61 (19), 6535-6546.

2. Winter, S.; Weber, E.; Eriksson, L.; Csöregh, I. New coordination polymer networks based on copper(II) hexafluoroacetylacetonate and pyridine containing building blocks: synthesis and structural study. *New J. Chem.* **2006**, 30 (12), 1808-1819.

3. McIlroy, S. P.; Cló, E.; Nikolajsen, L.; Frederiksen, P. K.; Nielsen, C. B.; Mikkelsen, K. V.; Gothelf, K. V.; Ogilby, P. R. Two-Photon Photosensitized Production of Singlet Oxygen: Sensitizers with Phenylene−Ethynylene-Based Chromophores *J. Org. Chem.* **2005**, 70 (4), 1134-1146.

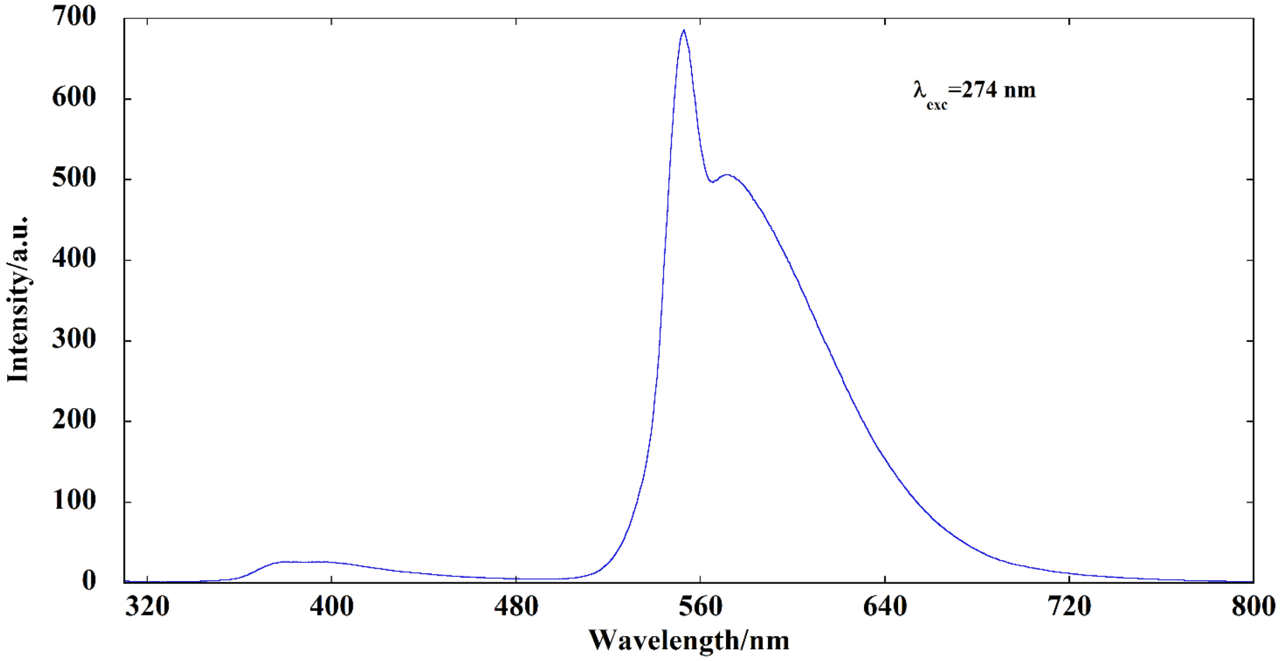

Figure S1. Fluorescence spectrum of a $AEPyI_2$ solution in water (1.2 mg/100 ml). The excitation wavelength used was 274 nm.

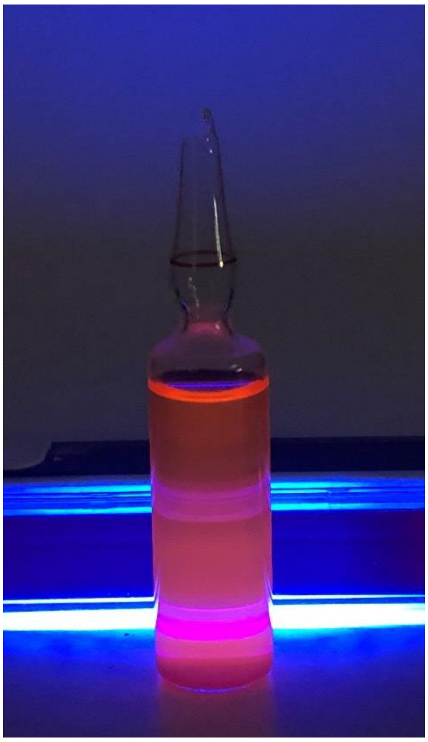

Fig. S2. Photo of a solution of $AEPyI_2$ in water excited with a 365 nm lamp.

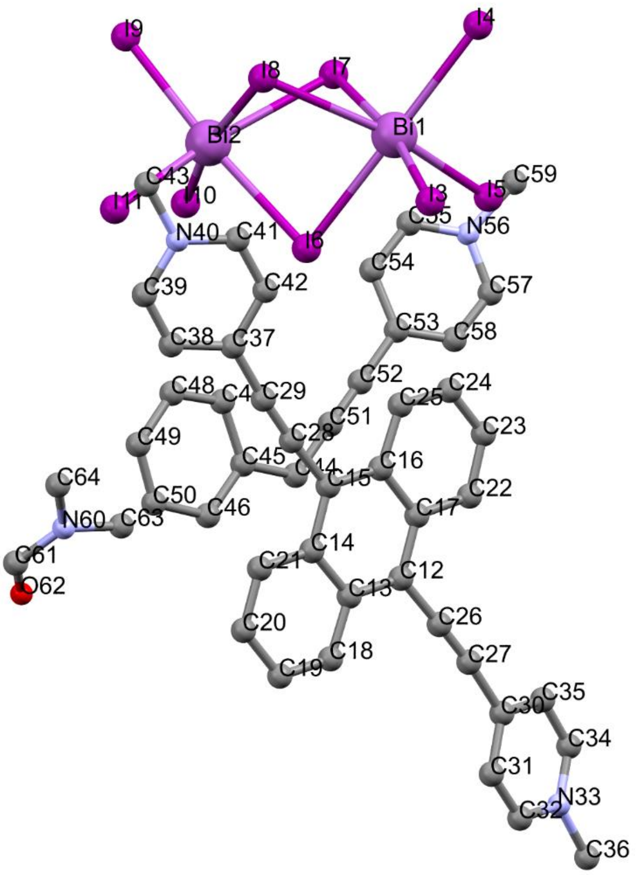

Figure S3. AEPyBiI stick-and-ball representation together with the labelling scheme, as found in the asymmetric unit. Hydrogens are omitted for clarity. Carbons are colored in dark grey, nitrogen in pale blue, iodine in dark purple, bismuth in lighter purple.

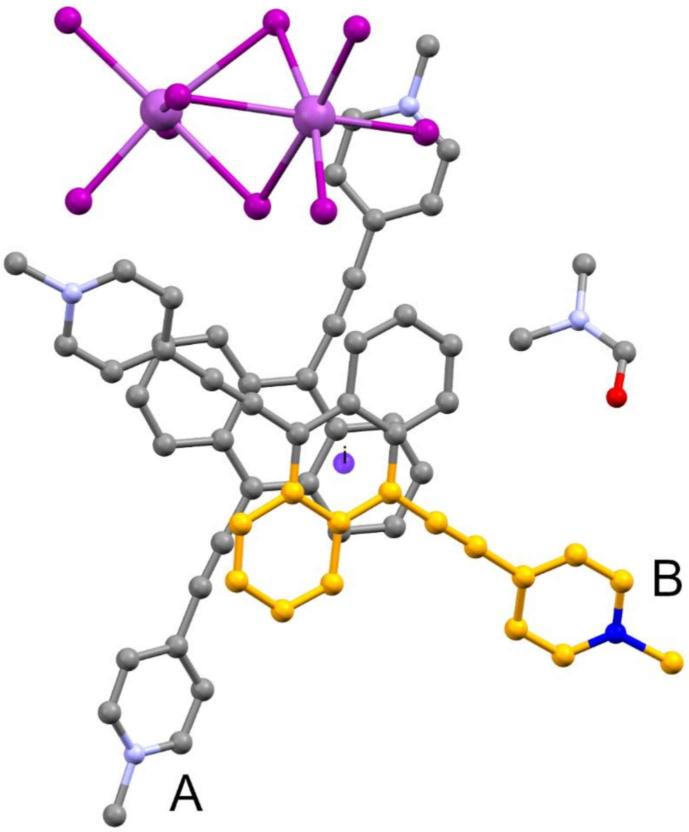

Figure S4: AEPyBiI including the symmetry generated half $AEPy^{2+}$ colored in orange. The inversion center lying on the anthracene moiety is also depicted as a sphere and colored in pale purple. The two different $AEPy^{2+}$, are labelled A and B according to the main text.

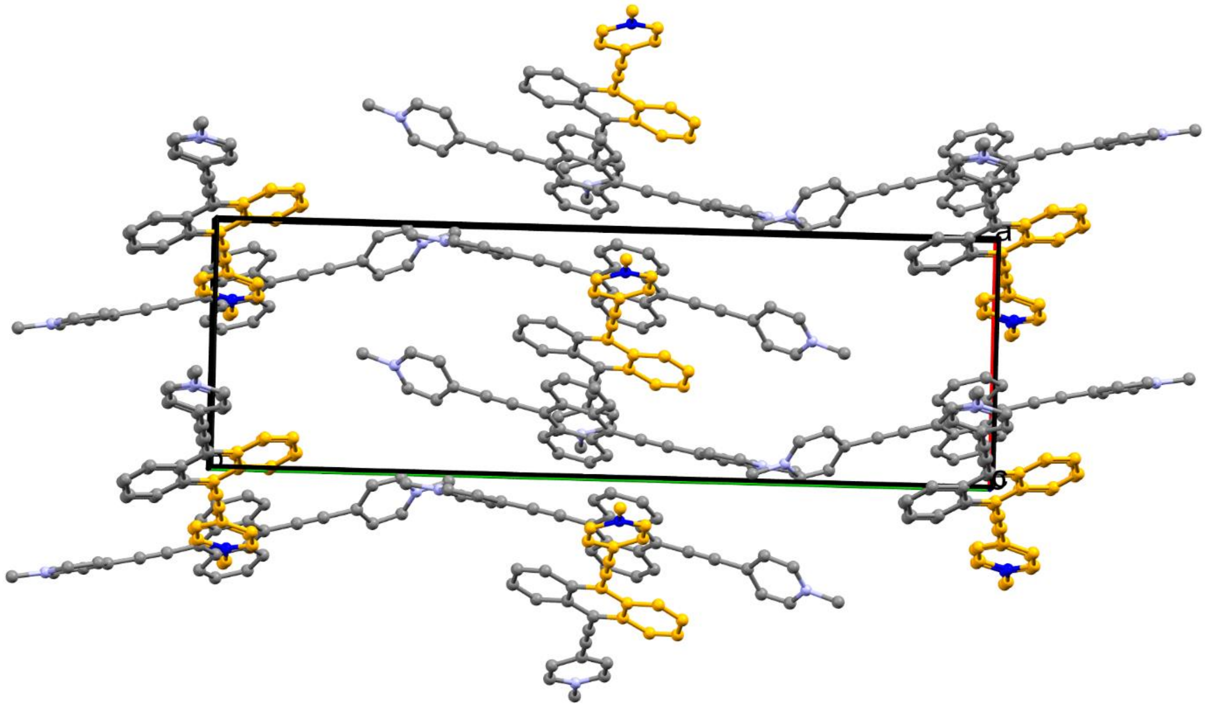

Figure S5: Stick-and-ball representation of the (ABA)(ABA) network as described in the main text, seen along the ***c*** axis. $AEPy^{2+}$ of type B are half colored in orange. $Bi_2I_9^{3-}$ and disordered DMF molecules are omitted for clarity.

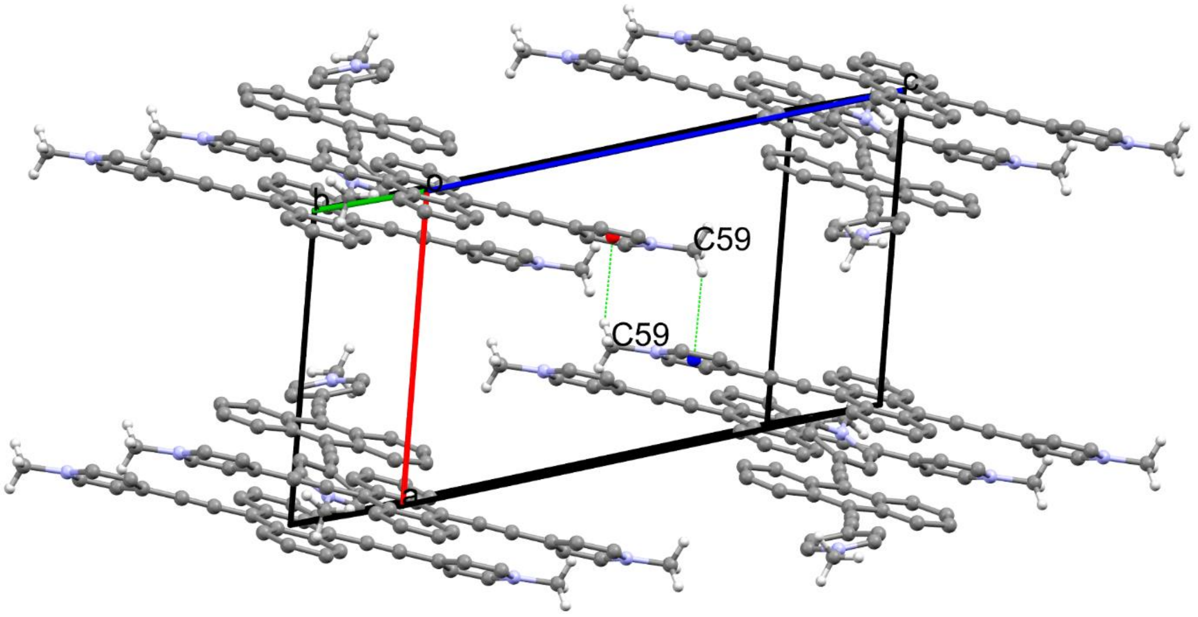

Figure S6: Stick-and-ball representation of the (AB)… $AEPy^{2+}$interaction network as described in the main text. Centroids of the pyridinium ions are represented as spheres colored in blue and red on neighboring cations. $Bi_2I_9^{3-}$ and disordered DMF molecules, as well as $AEPy^{2+}$ belonging to the (ABA)(ABA) network are omitted for clarity.

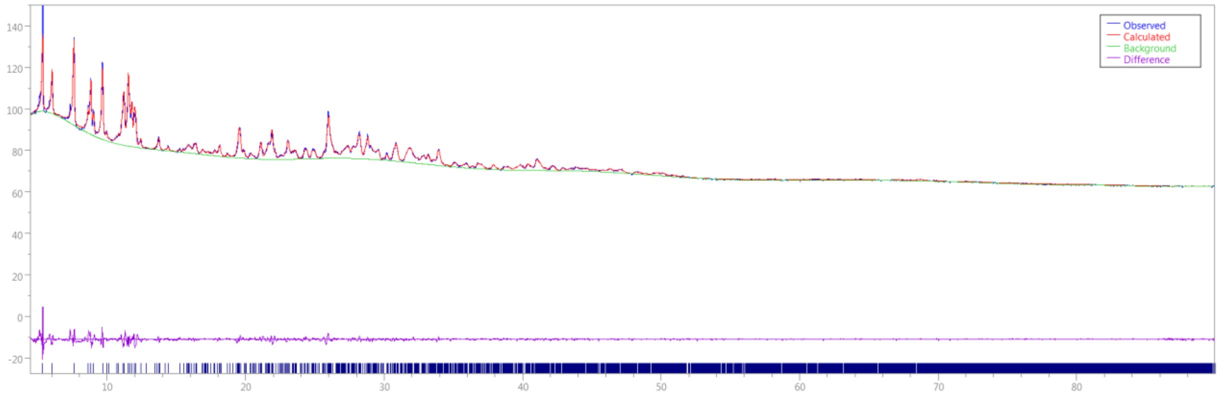

Figure S7. Le Bail fit of the experimental powder diffraction pattern using the structure obtained by single crystal data.

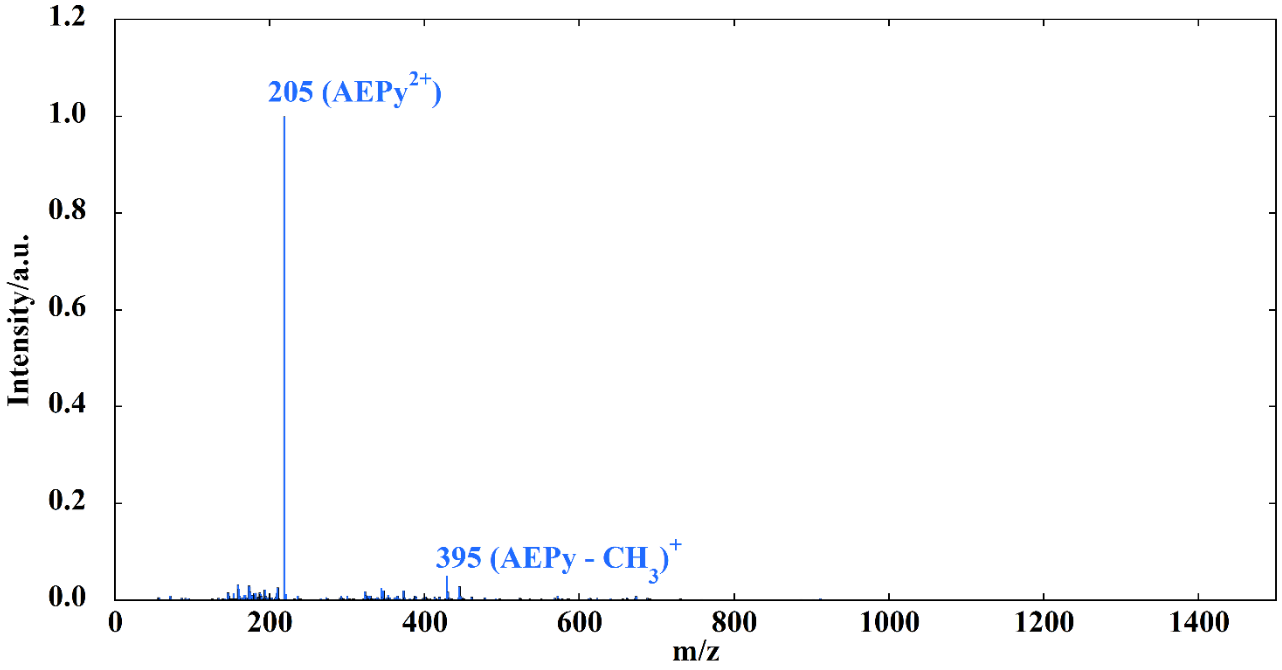

Figure S8. Positive ions ESI mass spectrum of AEPyBiI.

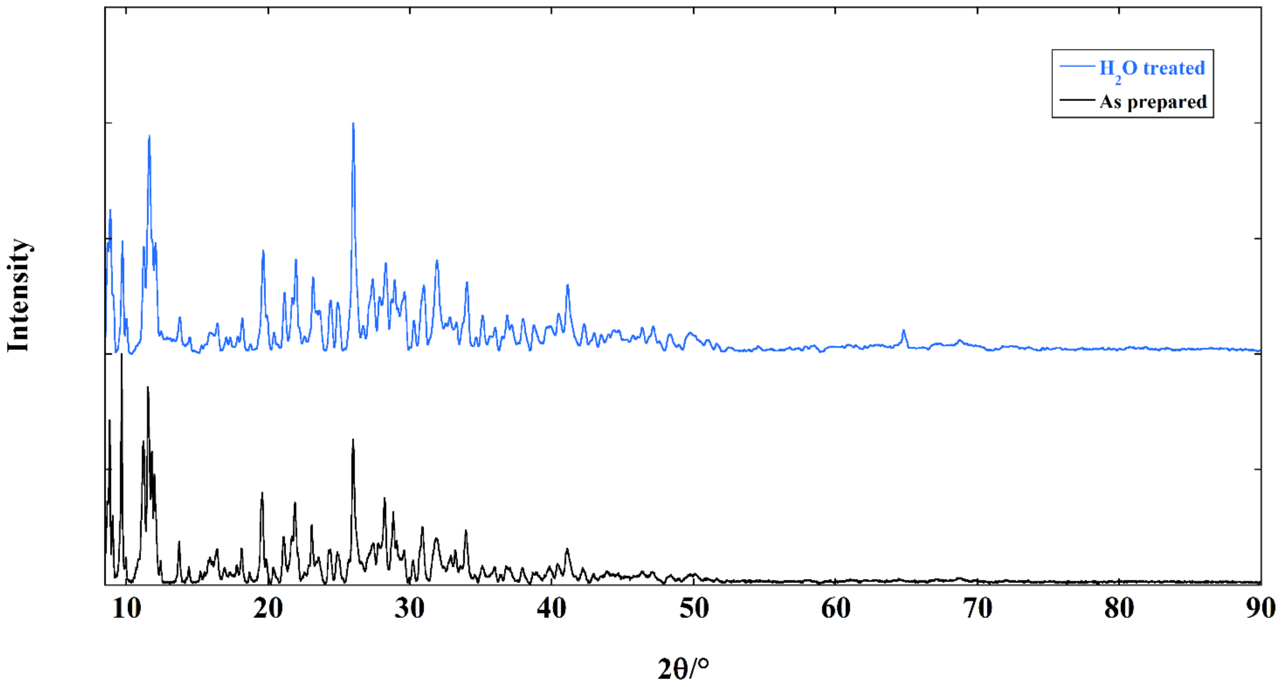

Figure S9. Background-subtracted XRD powder patterns of as prepared AEPyBiI and water treated AEPyBiI.

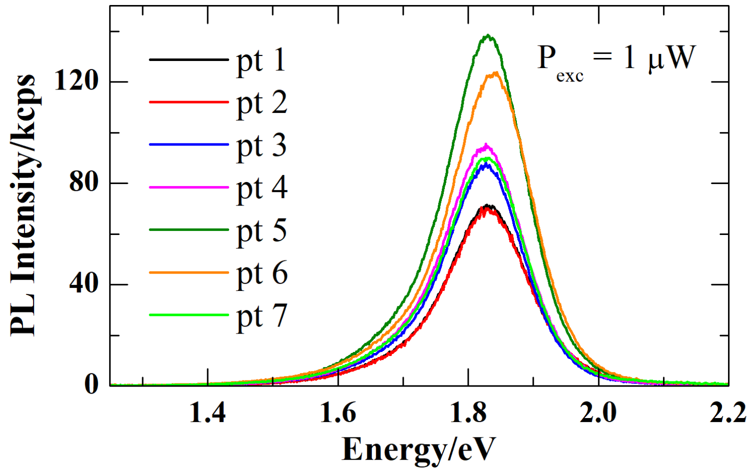

Figure S10. PL spectra of AEPyBiI acquired on 7 different points of the powder. The PL peak energy is rather homogeneous, varying between 1.826 eV and 1.837 eV. The intensity features moderate variations, considering that the measurements were taken on the powder. The spectrum shown in Fig. 9C of the main text was taken on point 1.

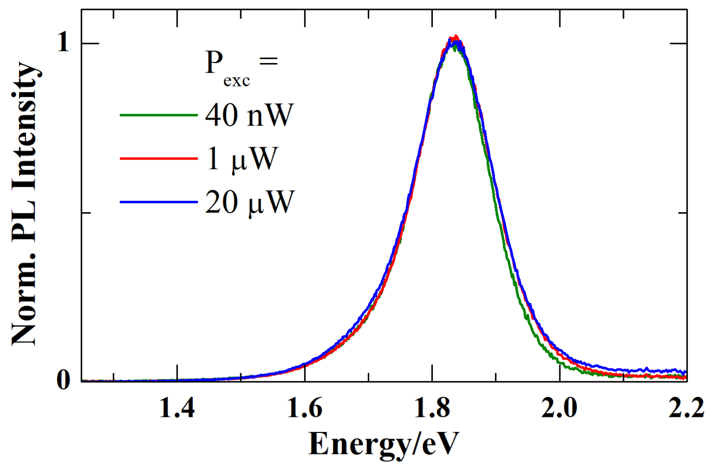

Figure S11. Normalized PL spectra of AEPyBiI acquired by exciting the sample with three different laser excitation powers P_{exc}, varying over about three orders of magnitude. The lineshape does not feature sizeable variations.

Bi2—I6	3.1545 (9)
Bi2—I8	3.2230 (10)
Bi2—I10	2.9907 (9)
Bi2—I11	2.9277 (12)
Bi2—I7	3.2954 (12)
Bi2—I9	2.9705 (10)
Bi1—I6	3.1606 (9)
Bi1—I8	3.3485 (9)
Bi1—I7	3.1572 (10)
Bi1—I4	3.0068 (10)
Bi1—I3	3.0141 (11)
Bi1—I5	2.8631 (9)

Table S1. Bismuth-iodine interatomic distances

I6—Bi2—I8	84.40 (2)
I6—Bi2—I7	81.46 (2)
I8—Bi2—I7	79.50 (3)
I11—Bi2—I10	98.73 (3)
I11—Bi2—I9	94.85 (4)
I9—Bi2—I10	91.89 (3)
I6—Bi1—I8	82.27 (2)
I7—Bi1—I6	83.58 (3)
I7—Bi1—I8	79.64 (3)
I4—Bi1—I3	94.56 (4)
I5—Bi1—I4	91.87 (3)
I5—Bi1—I3	92.73 (3)

Table S2. I-Bi-I angles formed with I atoms shared by the two octahedra and I-Bi-I angles formed with non-shared I atoms.
